# Matching Diabetes and Alcoholism: Oxidative Stress, Inflammation, and Neurogenesis Are Commonly Involved

**DOI:** 10.1155/2015/624287

**Published:** 2015-05-07

**Authors:** Jorge M. Barcia, Miguel Flores-Bellver, Maria Muriach, Javier Sancho-Pelluz, Daniel Lopez-Malo, Alba C. Urdaneta, Natalia Martinez-Gil, Sandra Atienzar-Aroca, Francisco J. Romero

**Affiliations:** ^1^Facultad de Medicina y Odontología, Universidad Católica de Valencia “San Vicente Mártir”, C/Quevedo 2, 46001 Valencia, Spain; ^2^Unidad Predepartamental de Medicina, Universitat Jaume I, 12071 Castellón de la Plana, Spain

## Abstract

Diabetes and alcohol misuse are two of the major challenges in health systems worldwide. These two diseases finally affect several organs and systems including the central nervous system. Hippocampus is one of the most relevant structures due to neurogenesis and memory-related processing among other functions. The present review focuses on the common profile of diabetes and ethanol exposure in terms of oxidative stress and proinflammatory and prosurvival recruiting transcription factors affecting hippocampal neurogenesis. Some aspects around antioxidant strategies are also included. As a global conclusion, the present review points out some common hits on both diseases giving support to the relations between alcohol intake and diabetes.

## 1. Introduction

In accordance with the International Diabetes Federation, latest diabetes statistics indicates that 382 million people are affected by diabetes in 2013. Alcohol use disorder (formerly called alcohol dependence or alcohol abuse) is the most abundant mental disorder in America, where approximately 14% of the population meets chronic alcoholic criteria during some period of their lives [1, 2]. It is closely related to several organic diseases and is involved in almost 50% of traffic accidents and the majority of homicides, suicides, and domestic violence cases [3]. Alcoholism and diabetes can affect several organs and systems. Alcohol exposure and diabetes can both be associated with cognitive impairment. In fact, diabetes-associated cognitive decline describes a state of cognitive impairment [4, 5]. Furthermore, diabetes increases the risk of Alzheimer's disease, vascular dementia, and any other type of dementia [6, 7]. Alcoholic dementia and ethanol related cognitive decline are also described [8–10].

The central nervous system (CNS) is especially vulnerable to oxidative damage as a result of its high oxygen consumption rate, its abundant lipid content, and relative paucity of antioxidant enzymes as compared to other tissues. Neural tissue is particularly sensitive to oxidative insults; in fact reactive oxygen species (ROS) are involved in many diseases finally affecting the CNS [11, 12]. Under these two pathological conditions, cellular stress triggers mitochondrial oxidative damage, which may result in apoptosis and/or necrosis [13–15].

## 2. Diabetes and Ethanol Exposure Promote Oxidative Stress: AGE and Aldehydes

It is well documented that diabetes and alcohol exposure are accompanied by alterations in the redox status. Hyperglycemia and ethanol exposure reduce antioxidant levels and increase the production of free radicals with subsequent activation of redox-sensitive genes [15–21].

Glutathione system, including both reduced and oxidized forms (GSH and GSSG, resp.) and the glutathione peroxidase enzyme (GPx), is one of the most important cellular antioxidant defense systems due to its capacity of trapping ROS. GSH is ubiquitously present including the central nervous tissue [22]. It is well documented that hippocampal GSH/GSSG ratio and GPx activity are significantly reduced in diabetes or by ethanol exposure [15, 18–21, 23–26]. Interestingly, this decrease can be prevented by antioxidant treatment in both cases [17, 19, 26, 27], indicating that ROS could be at least partially responsible of the GSH content and GPx activity decreases.

ROS usually react with lipids, a process known as lipid peroxidation. Since the CNS is particularly rich in fatty acids, this peroxidation results in aldehyde production, for example, malondialdehyde (MDA) and 4-hydroxyalkenals such as 4-hydroxy-nonenal (4-HNE). These end-products may create adducts leading to DNA alterations (for a review see [28]) and protein modifications. These facts can produce enzymatic activity alterations or lack of DNA sequence-recognizing, among other side effects. In this way, 4-HNE inhibits Akt1 by competitive inhibition of ATP at the kinase domain of ATP binding sites resulting in increased ROS levels and cell death [29]. ROS production decreased antioxidant defense and increased lipid peroxidation and membrane degeneration, leading to cellular damage/death in diabetes or ethanol exposure [30–32]. In fact, mitochondrial dysfunction and reduced ATP biosynthesis have been implicated in diabetes and ethanol exposure [15, 33]. Accumulation of lipid peroxidation products such as 4-HNE and MDA in mitochondria has also been reported in diabetic patients, possibly causing further damage to mitochondrial genetic and metabolic systems [34, 35]. In this sense, recent data from our laboratory indicate a marked increase in 4-HNE aggregates after ethanol exposure in a human retinal pigment epithelial cell line (ARPE-19) accompanied by mitochondrial degeneration and mitophagy [36].

One of the main questions concerning ethanol and diabetes is the one related to how hyperglycemia and ethanol exposure exert primarily their negative effects. Evidences are accumulating pointing to the role of oxidative stress and proinflammatory mechanisms on both pathological conditions, but little is known about the molecular mechanisms.

Circulating proteins or lipids can be modified by circulating sugars and even aldehydes such as acetaldehyde [37, 38]. These products are known as advanced glycation end products (AGEs) [39, 40]. AGEs can produce ROS and AGEs can bind to specific cell surface receptors (RAGE) [40, 41]. AGEs produce ROS via NAD(P)H oxidase and also activate nuclear factor kappa B (NF-*κ*B) [20, 42]. Further support for this idea is the finding of increased expression of serum AGEs in alcoholic patients and RAGE in the prefrontal cortex of human alcoholic patients [43, 44]. In fact, some toxic effects promoted by experimental diabetes are increased by ethanol administration [45].

## 3. Cytochrome p450 2E1 (CYP2E1) Is Commonly Implicated

Another relevant enzyme related to diabetes and ethanol exposure is cytochrome P450. This is a family of enzymes involved in the oxidative metabolism of both endogenous and xenobiotic products [46–48]. Particularly involved in EtOH oxidation, CYP2E1 isoform assumes an important role in metabolizing ethanol being considered as a major component of the microsomal ethanol-oxidizing system (MEOS) [49, 50].

Increased expression of hepatic CYP2E1 in human or experimental diabetes and alcohol abusers has been reported [51–54]. Ethanol-induced CYP2E1 gene transcription is ROS-mediated and therefore CYP2E1 induction is accompanied by more ROS production and vice versa [55]. In fact, diallyl sulphide (DAS), a competitive inhibitor of CYP2E1, can directly block ROS production and also inhibit CYP2E1 induction [55]. A parallel increase of CYP2E1 expression and ROS production in brain, kidney, and liver of streptozotocin-induced diabetic rats has been also reported and these increases can be blocked by ascorbic acid [56, 57]. So CYP2E1 and ROS are reciprocally modulated in diabetes and ethanol exposure. Interestingly, NF-*κ*B regulates CYP2E1 expression by different ways, being implicated in several diseases including diabetes [58]. Considering that CYP2E1 is not only found in liver, but can be also considered almost ubiquitously present, the possibility that an ethanol extrahepatic detoxifying activity could be present in other tissues, including the brain, must be taken into account.

## 4. Hippocampal Neurogenesis Is Affected in Diabetes and Ethanol Exposure

Hippocampus is part of the temporal lobe being considered crucial for several cognitive processes as well as for spatial navigation and memory processing [59]. Hippocampus is particularly affected in several diseases, for example, Alzheimer's disease and diabetes, or in alcohol use disorder [60, 61]. One of the particularities of this neural structure is that referred to neurogenesis. Hippocampus shares this privilege with the subventricular zone (SVZ). Concretely, the subgranular zone (SGZ) of the dentate gyrus presents a discrete stem cell population [62]. Newborn subgranular cells differentiate to neurons being finally integrated into the granular cell layer (GCL) thickness [63]. Although the concrete function or meaning of this neuronal incorporation still remains unclear [64], the inhibition of stem cell proliferation or the death of newborn cells is accompanied by an impairment of hippocampal-dependent functions. Focusing on diabetes and alcohol consumption, it is well established that both conditions can affect cognitive processes and neurogenesis.

A classical experimental task evaluating hippocampal-dependent functions is the popular Morris water maze test, based on the location of an underwater hidden platform. Independently of the swimming speed, animals treated with ethanol exhibit more latency time to find the platform. In other words they need more time to learn. This task is considered a good tool assessing hippocampal function. Giving support to this phenomenon is the finding that hippocampal long term potentiation (LTP) is also disrupted. Experimental LTP is considered as a good tool to approach synaptic plasticity and therefore learning processes [65].

Interestingly the simultaneous triad consisting of neurogenesis reduction, spatial navigation impairment, and hippocampal LTP disruption is usually present in diabetes and ethanol exposure [17, 19, 66–68], suggesting close relationship among them. Furthermore, all these alterations can be prevented by antioxidant treatment adding more evidence to this relationship [19, 68]. Although it seems clear that ROS are relevant in these processes, evidences are also accumulating pointing to the glucocorticoid pathway, since it has been demonstrated how those hippocampal alterations (including neurogenesis, LTP, and water maze test) in type I and II diabetes are normalized when glucocorticoid levels are in a physiological range [69]. The negative effects of high glucocorticoid levels on hippocampal neurogenesis and synaptic plasticity are well documented [69–72]. Ethanol exposure also alters the hypothalamic pituitary axis (HPA): in a rat model of binge drinking, increased corticosterone levels led to dentate gyrus degeneration and this phenomenon seemed to be mediated by type II glucocorticoid receptor [73], in agreement with that occurring in diabetes.

## 5. CREB, NF-*κ*B, and Nrf2 Are Altered in Diabetes and Alcohol Exposure

NF-*κ*B and cAMP responsive element-binding protein (CREB) are both transcription factors, respectively, related with proinflammatory and prosurvival genes.

CREB-related genes are transcribed by the phosphorylation of CREB (p-CREB). Those CREB-related genes encoding neurotrophins are generally related to neuronal survival, cell death protection, and neural plasticity, as are the brain derived neurotrophic factor (BDNF), B-cell lymphoma 2 (Bcl-2) protein, nerve growth factor (NGF), and vascular endothelial growth factor (VEGF) genes [74, 75].

CREB/p-CREB signaling has been related to neurite out-growth, learning-memory and LTP [76–78].

Although NF-*κ*B is a transcriptional factor typically implicated in inflammatory and immune responses [79], NF-*κ*B is also involved in neuroprotection, being implicated in cell division, synaptic plasticity, neurite growth, and formation of functional dendritic spines [79–83].

NF-*κ*B can be activated by oxidative stress or other signals such as cytokines or glutamate [79, 82, 84]. In fact, NF-*κ*B is induced in several conditions such as neurodegenerative diseases and brain injury [81, 85, 86].

As indicated above, ethanol exposure and diabetes promote oxidative stress in the CNS and particularly in the hippocampus. At the same time both, ethanol exposure and diabetes, decrease hippocampal CREB phosphorylation [18, 87, 88] while they increase NF-*κ*B activity [17, 18, 88]. Additionally, deregulation of inflammatory genes has been found in hippocampi of alcoholic patients [89] and furthermore prefrontal cortices from alcoholic patients also show NF-*κ*B/p50 downregulation [90].

Ethanol-induced p-CREB decrease is accompanied by reductions in BDNF gene expression contributing to hippocampal neurotoxicity [91]. In fact, hippocampal BDNF is also decreased in a type 2 diabetes animal model and it is associated with hippocampal dependent memory impairment [92, 93]. Furthermore, caffeine and huperzine restore hippocampal-dependent memory impairment via BDNF [94, 95].

Nuclear factor (erythroid-derived 2)-like 2 (Nrf2) binds to the antioxidant response element (ARE) sequence promoting the transcription of antioxidant-related proteins as NAD(P)H quinone oxidoreductase 1, gluthatione S-transferase, or GPx among others [96]. According to Cederbaum [97] Nrf2 plays a key role in the adaptive response against increased oxidative stress caused by CYP2E1 in HepG2 cells. Nrf2 activation inhibits NF-*κ*B activation by reducing ROS and by inhibiting I*κ*B degradation [97, 98]. NF-*κ*B also binds to ARE sequence competing with Nrf2 [99] and therefore the result of this balance may lead to proinflammation or antioxidant defense responses.

Focusing on hippocampal neurogenesis, a positive regulation of cell proliferation has been demonstrated by p-CREB in the SGZ [100]. Stressing the role of CREB in this process, hippocampal neurogenesis is also a GABA-CREB dependent process regulating maturation and survival of newly generated subgranular neurons [101]. All these evidences give support to the possibility that ethanol and diabetes may reduce hippocampal neurogenesis in the same way.

According to previous reports [80–82], NF-*κ*B may play different roles in hippocampal neurogenesis depending on the cell stage. In neuronal progenitor cells (NPC) it regulates axogenesis and maturation, whereas in mature granule cells (MGC) it regulates neuroprotection and synaptic transmission. In fact NF-*κ*B inhibition promotes dentate gyrus atrophy [80].

How some genes related to neurogenesis and synaptic plasticity are altered by diabetes as well as how dietary supplementation with resveratrol normalized them has been described. Furthermore, proinflammatory genes from the Jak-Stat pathway are also repressed after resveratrol administration [102]. In this sense, it is interesting to note that resveratrol acts as a mixed agonist/antagonist for estrogen receptors [103] that induces Bcl-2 expression via CREB and Akt phosphorylation [104], so its action is not solely by direct ROS trapping [105].

Regarding the CREB-related pathway, it has been shown that Nrf2 and NF-*κ*B compete for the transcriptional coactivator CREB-binding protein (CBP) [106]. Additionally, CBP/p300 complex is a histone acetyltransferase (HAT) associated with CREB, Nrf2, NF-*κ*B, and AP-1 gene transcription [107]. However, CBP is also associated with glucocorticoid receptors [108], resulting in HAT activity inhibition with subsequent transcriptional blockade of the genes above; interestingly, curcumin has been described as an inhibitor of CBP/p300 [109]. Several data are focusing on the neuroprotective role of curcumin improving hippocampal neurogenesis in some animal models such as ischemic brain injury [110] and chronic stress [111]. Curcumin alleviates neuropathic pain by reducing spinal BDNF and cox-2 gene expression in a rat model of neuropathic pain [112], and interestingly the Akt/Nrf2 pathway is involved in neuroprotection after brain ischemia [113]. However and surprisingly, hippocampal neurogenesis inhibition, under chronic stress conditions, can be reversed by curcumin via increasing BDNF [110].

High glucose and ethanol exposure promote glucose misbalance, HPA deregulation with metabolic and molecular changes affecting different systems but particularly CNS. ROS and oxidative stress misbalance are present as common background feature and may be the reason of the similarities observed between high glucose and ethanol exposure such as the presence of AGE, good outcome to antioxidant strategies, and hippocampal neurogenesis inhibition. Since alcoholism and diabetes in humans are long lasting diseases with a “waveform-like” behavior in terms of intermittent relapse-abstinence, binge or chronic drinking, randomly insulin control, or uncontrolled glycemia, it seems plausible that alcoholism and diabetes may lead to neuroadaptive changes.

**Figure 1:**
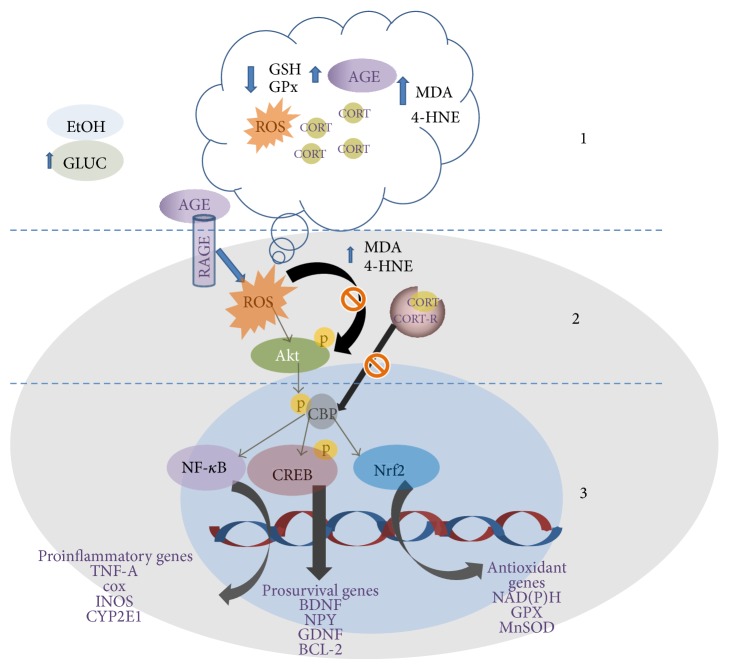
*Scheme summarizing common pathways implicated in oxidative stress, inflammation, and antioxidant responses.* 1. Circulating ethanol and high glucose levels lead to ROS production, antioxidant defense decay, AGE, and aldehyde production (4-HNE, MDA). Alterations on the hypothalamic pituitary axis (HPA) produce glucocorticoid release. 2. RAGE activation and ROS activate Akt but increased levels of 4-HNE; MDA can block Akt phosphorylation. 3. CBP is an acetyl transferase (HAT) that depends on p-Akt. CBP is necessary for p-CREB, Nf-*κ*B, and Nrf2 allowing transcription of proinflammatory or anti-inflammatory genes. Glucocorticoid receptors inhibit CBP activity blocking transcription. Since Nf-*κ*B, p-CREB, and Nrf2 compete for CBP the balance on proinflammation versus anti-inflammation transcription is compromised. GSH: reduced glutathione; NF-*κ*B: nuclear factor kappa B; MDA: malondialdehyde; ROS: reactive oxygen species; MnSOD: manganese superoxide dismutase; TNF*α*: tumor necrosis factor alpha; COX: cyclooxygenase; iNOS: inducible nitric oxide synthase; GPx: glutathione peroxidase; BDNF: brain-derived neurotrophic factor; NGF: nerve growth factor; Bcl-2: B-cell lymphoma 2; CORT: corticosteroid; CORT-R: corticosteroid receptor; P-CREB: phospho-cAMP response element-binding; CBP: CREB-binding protein; Nrf2: nuclear factor erythroid-derived 2; 4-HNE: 4-hydroxynonenal; Akt: protein kinase B; CYP2E1: cytochrome P450 2E1; RAGE: receptor for advanced glycation end products.

Transcription signaling for CREB, Nrf2, and NF-*κ*B dependent genes is commonly regulated by CBP activity and furthermore the competition for DNA sequences might explain the outcome of these diseases. Exposure to high glucose levels or ethanol seems to act on a similar way activating similar genes and some antioxidants as curcumin or resveratrol are effective by activating up-stream molecules modulating those aforementioned genes. ROS inhibition significantly alleviates many of the deleterious effects promoted by hyperglycemia or ethanol exposure. So the combination of direct ROS scavengers and antioxidants as curcumin or resveratrol can not only block cellular damage but also modulate gene transcription activity (see Figure 1).

## 6. Final Summary

Several cellular and molecular alterations are shared during ethanol exposure or under diabetic conditions. Hippocampal neurogenesis is negatively affected and CREB/p-CREB, NF-*κ*B, Nrf2, and CYP 2E1 are involved and modified in both situations. Oxidative stress is a key factor of these situations, since the addition of antioxidants (trapping ROS) can reverse or normalize all these aforementioned alterations. Furthermore, evidences are also focusing on downstream effects of antioxidants in terms of activation-repression of transcription factors such as CREB or Nrf2, making of antioxidant therapies a good tool against neural and functional alterations in diabetes and alcohol-related memory or cognitive impairments. Diabetes and chronic ethanol exposure affect different organs and systems and some of their negative effects can be improved or prevented by antioxidant therapies. Knowing how antioxidants can prevent those negative effects or elicit cellular protective responses may be helpful for future development of new therapeutic tools against these two diseases affecting millions of people worldwide.

Additionally, experimental and clinical studies strongly indicate a close relationship between alcohol intake and risk of diabetes development [114–117]. Considering all data available, and reviewed herein (molecular signaling, genes, and transcription factors), it seems plausible that all overlapping mechanisms of alcohol intake and diabetes could explain this relationship.
